# Occurrence and Risk Assessment of Pyrrolizidine Alkaloids in Spices and Culinary Herbs from Various Geographical Origins

**DOI:** 10.3390/toxins12030155

**Published:** 2020-03-01

**Authors:** Florian Kaltner, Michael Rychlik, Manfred Gareis, Christoph Gottschalk

**Affiliations:** 1 Chair of Food Safety, Faculty of Veterinary Medicine, Ludwig-Maximilians-University of Munich, Schoenleutnerstr. 8, 85764 Oberschleissheim, Germany; 2 Chair of Analytical Food Chemistry, Technical University of Munich, Maximus-von-Imhof-Forum 2, 85354 Freising, Germany

**Keywords:** pyrrolizidine alkaloids, spices, culinary herbs, food safety, contamination, LC-MS, risk assessment

## Abstract

Pyrrolizidine alkaloids (PA) and their *N*-oxides (PANO) are a group of toxic secondary plant metabolites occurring predominantly as contaminants in (herbal) teas, honeys and food supplements, as well as in spices and culinary herbs. Depending on the botanical origin of the contaminating plant, the pattern of PA/PANO can strongly vary within a sample. The current study aimed to broaden the existing data on the occurrence of PA/PANO in spices and culinary herbs. For this, 305 authentic samples covering 15 different matrices mainly harvested in 2016 or 2017 and originating from 36 countries were investigated for the presence of 44 PA/PANO. Fifty-eight percent of the samples contained at least one PA/PANO. The average sum content over all samples was 323 µg/kg (median of 0.9 µg/kg, 95% percentile of 665 µg/kg). The highest amount of 24.6 mg/kg was detected in an oregano sample. Additionally, conspicuous analyte patterns were discovered in samples from similar cultivation regions, indicating related botanical sources of PA/PANO contaminations. Particularly, oregano and cumin from Turkey often contained high amounts of PA/PANO. The results were used to assess the acute and chronic health risks related to PA/PANO intake via spices and culinary herbs, indicating a potential health risk in particular for adults and children with high consumption or when considering worst-case contamination scenarios of a sum content of 5500 µg/kg.

## 1. Introduction

Pyrrolizidine alkaloids (PA), including their corresponding *N*-oxides (PANO), are a group of toxic secondary metabolites presumably distributed in more than 6000 plant species worldwide, especially occurring in the families Asteraceae (tribus Senecioneae and Eupatorieae), Boraginaceae, Apocynaceae (tribus Echiteae) and Fabaceae (genus *Crotalaria*) [1,2]. More than 660 PA/PANO were identified so far, occurring in presumably 3% of all flowering plants in varying compositions [3,4]. The 1,2-unsaturated types of PA/PANO, mainly of the retronecine-, heliotridine- and otonecine-type, are known to be toxic for humans and animals [5]. Cytochrome-P450 monooxygenase enzymes in the liver can transform these compounds into highly reactive pyrrole esters, which are able to bind to proteins and DNA and cause dysfunctions of affected proteins and exhibit genotoxic properties [6]. Adverse health effects of an acute PA/PANO intoxication are primarily characterised by the hepatic sinusoidal obstruction syndrome, formerly known as veno-occlusive disease. A long-term intake of small amounts of PA/PANO is associated with chronic diseases such as liver cirrhosis, cancer or pulmonary arterial hypertension [7,8,9].

PA/PANO can contaminate food and feed. Especially honey, (herbal) teas and herbal food supplements are well-known sources of PA/PANO exposure to humans [10]. A contamination of these products can occur in different ways: the contamination of honey is caused by bees collecting pollen and, in particular, nectar from PA/PANO producing plants [11]. Contrary to this, a contamination of herbal products with PA/PANO was assumed to be exclusively caused by impurities or unintended co-harvesting of the aimed culture plants and weed plants containing these toxins. Recently published data also report the horizontal transfer and exchange of PA/PANO between living plants via the soil as an unconventional and yet disregarded source of contamination [12].

Although the toxic effects of PA/PANO are known and thus contents in relevant food and feed matrices have been monitored for years, regulatory limits have not been established in the European Union so far. The risk of a PA/PANO exposure was assessed by the European Food Safety Authority (EFSA), using the margin of exposure (MOE) approach based on a Benchmark Dose Lower Confidence Limit 10% (BMDL_10_) of 237 µg/kg body weight/day (BW/d) for the PA/PANO sum [10]. Until now, only a maximum content of 100 mg/kg of PA/PANO-containing *Crotalaria* spp. for animal feed materials and compound feed was set in Directive 2002/32/EC [13]. Regulatory limits for these toxic contaminants in food are still discussed and may be implemented in Commission Regulation (EC) No. 1881/2006 within the next year [14]. However, PA/PANO are known to be genotoxic and carcinogenic, thus their occurrence in food and feed supply chains should generally be kept as low as reasonably achievable (ALARA principle) [15].

PA/PANO can also occur in spices and culinary herbs, as these products are subject of the same way of contamination already known for (herbal) tea plants [16]. Considering the few available results, contaminated spices and herbs presumably may greatly contribute to the overall exposure of consumers to PA/PANO [17]. Thus, the aim of the present study was to expand knowledge on the occurrence of PA/PANO in different types of spices and culinary herbs to identify potential sources with relevance for food safety. Therefore, we analyzed 305 samples of 15 different varieties from numerous geographical origins from all over the world for the presence of 44 PA/PANO compounds. The results were evaluated with respect to their PA/PANO patterns, geographical and botanical origin. Additionally, data were used to perform a risk assessment for PA/PANO in spices and culinary herbs, using exemplary recipes and consumption data from nutritional studies for calculating exposure of adults and children.

## 2. Results

### 2.1. Occurrence of PA/PANO in Spices and Culinary Herbs

Samples were investigated for the presence of PA/PANO using an approach published earlier [18]. The analytes were extracted under acidic conditions and the raw extracts were further cleaned using polymeric cation exchange solid phase extraction (PCX-SPE) cartridges. The compounds were detected via liquid chromatography tandem mass spectrometry (LC-MS/MS). Quantification of PA/PANO was performed using matrix-matched external calibration.

**Figure 1:**
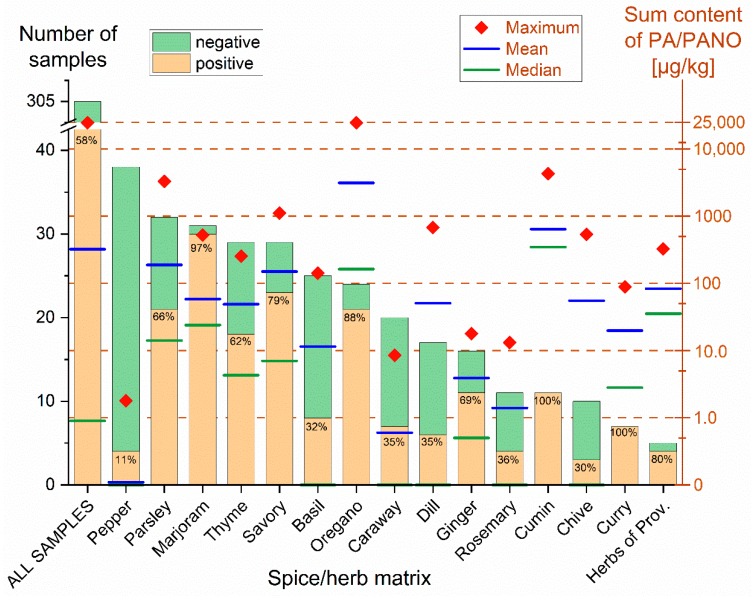
Prevalence and contents of pyrrolizidine alkaloids (PA) and PA *N*-oxides (PANO) in spice and culinary herb samples. The number of investigated samples of each matrix can be derived from the left Y-axis. The percentage on each bar represents the prevalence (positive samples/all tested samples) in the respective matrix (X-axis). Median, average and maximum PA/PANO sum contents can be taken from the right Y-axis. The median bar of spices and herbs with <50% positive samples is 0 µg/kg and thus not drawn. For the sake of clarity, the sum contents were displayed in a logarithmic manner.

Among 305 samples tested, 178 (i.e., 58.4%) were found to contain at least one PA/PANO above the limit of detection (LOD) (Figure 1). For calculation reasons, contents <LOD were further treated as 0.0 µg/kg and contents between LOD and the limit of quantification (LOQ) were calculated with 0.5 times the LOQ. High prevalence of PA/PANO was determined in marjoram, savory, oregano, cumin, curry and herbs of Provence, all showing contamination rates of more than 79%. Showing 11% positive samples, the smallest prevalence was obtained for pepper, and also basil, caraway, dill, rosemary and chive showed smaller contamination rates ranging from 30% to 36%. The remaining matrices, namely parsley, thyme and ginger showed a higher prevalence with 62% to 69% of the samples containing PA/PANO.

The sum contents in positive samples were markedly different and ranged from 0.1 µg/kg up to a maximum of 24,600 µg/kg (for exact contents, see Table S1). The median content over all samples tested was 0.9 µg/kg with an average content of 323 µg/kg and a 95% percentile of 665 µg/kg. The smallest average content was obtained for pepper (0.1 µg/kg). Further matrices with small average contents were thyme, dill, chive, marjoram and herbs of Provence, ranging from 49.1 to 83.4 µg/kg. High average contents above 100 µg/kg were determined in the matrices parsley (189 µg/kg), savory (150 µg/kg), cumin (641 µg/kg) and oregano (3140 µg/kg). In addition to their high amounts of PA/PANO, the two latter ones also showed comparably high median contents of 348 and 163 µg/kg, respectively.

### 2.2. Contents of PA/PANO in Samples from Different Countries

Notably, samples originating from South America were contaminated with levels near or below LOD (Table S1). Only three out of 12 samples (25.0%) were found to be positive, showing only small sum contents up to 41.6 µg/kg in one oregano sample from Chile. In total, 34 out of 62 samples (54.8%) from Africa contained at least one PA/PANO, and the highest amount was determined in a dill sample from Tunisia, containing 681 µg/kg. The contamination rate of samples from Asia, including the Middle Eastern States Israel, Turkey and Syria, was comparable with the one of Africa (36 positive samples of 71, 50.7%). Contrary to this result, high amounts in individual samples were mostly detected in Asian samples, with 10 of 36 positive samples containing more than 400 µg/kg (1000 µg/kg for dried marjoram and dried oregano; these levels represent the currently discussed maximum levels for dried herbs for implementation in Commission Regulation (EC) No. 1881/2006 [14]). The maximum sum content in a sample from Asia was 24,600 µg/kg. Samples originating from Europe showed the highest prevalence, as PA/PANO were found in 76 of 118 spices and culinary herbs (64.4%). Here, the maximum content was 11,030 µg/kg in an oregano sample originating from Greece. With amounts of 11 of 76 positive samples exceeding 400 µg/kg, the detected sum contents in European samples were lower compared to those from other continents.

In Table 1, samples with contaminations of PA/PANO exceeding the discussed maximum level of 400 µg/kg are summarized. Remarkably, nine of the 24 samples were oregano, and ten were cultivated in Turkey. In twelve of 24 samples, a heliotrine (Ht)-type PA/PANO was the predominant compound (europine/-NO, heliotrine/-NO and lasiocarpine-*N*-oxide), and 22 of them were PANO. This evaluation revealed high contributions of single PA/PANO ranging from 26% to 86% of the total content in a sample.

### 2.3. Patterns of PA/PANO in Samples from Various Geographical Origins

In total, 33 PA/PANO out of the tested set of 44 analytes were identified in the samples (Table S1). The most abundant PA/PANO were from senecionine (Sc)-type (49%), typically originating from species of the Asteraceae family. Within the covered analytes from the Sc-type, merenskine, merepoxine, sceleratine or their *N*-oxides were identified in no sample.

The next most frequently occurring group of analytes were from Ht-type (28%, from Boraginaceae) and lycopsamine (Ly)-type (23%, from Boraginaceae). In case of Ly-group, namely the *N*-oxides of acetylintermedine and acetyllycopsamine were not detected to occur in the samples. 

Contrary to the Sc-, Ht- and Ly-type PA/PANO, no analytes belonging to the monocrotaline (Mc)-type (trichodesmine, monocrotaline and its *N*-oxide, from Fabaceae) were determined in the investigated set of 305 samples. Among positive samples, the five most frequently occurring individual PA/PANO were lasiocarpine (32.1%, Ht-type), senecionine-NO (31.8%, Sc-type), integerrimine-NO (29.2%, Sc-type), senecionine and senkirkine (both 25.9%, Sc-type). Contrary to that, the quantitatively predominant analytes were the *N*-oxides of europine, lasiocarpine and heliotrine, all belonging to Ht-type PA/PANO.

**Figure 2:**
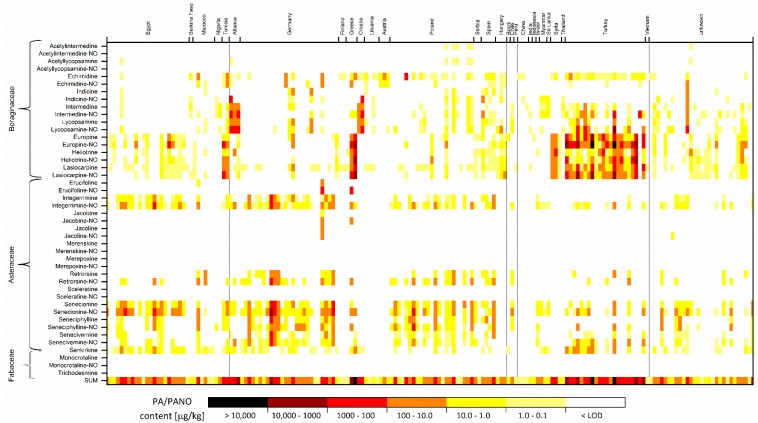
Heat map plot of amounts of pyrrolizidine alkaloids (PA) and PA *N*-oxides (PANO) in positive samples (*n* = 178). Analytes were grouped according to their botanical family of origin and countries of sample origins were arranged according their related continent (Africa, Europe, South America, Asia, unknown).

The distribution of individual analytes in positive samples originating from different countries and continents showed different predominant groups of PA/PANO (Figure 2). Sc-type compounds occurring in species of the Asteraceae family, covering retrorsine, senecionine, senecivernine, seneciphylline, integerrimine and their *N*-oxides as well as senkirkine, occurred more often in samples from Northern Africa and Central Europe, namely Germany and Poland. Contrary to that, Ly-type PA/PANO were predominantly found in samples originating from the Balkan region, with high percentages of indicine, lycopsamine and intermedine (+ *N*-oxides) in positive samples from Croatia and Albania. In spices and culinary herbs from Greece and, in particular, from Turkey, PA/PANO belonging to the Ht-type were predominant, and high amounts of europine, heliotrine and lasiocarpine as well as their *N*-oxides were detected.

### 2.4. Acute Health Risk Assessment of PA/PANO in Spices and Culinary Herbs

The determined levels of PA/PANO contamination in the investigated spices and culinary herbs were assessed for their risk regarding the health of consumers. The risk of an acute non-carcinogenic health damage was assessed on the basis of a health-based guidance value (HBGV) which has been derived from a No-Observed-Adverse-Effect-Level (NOAEL) of 10 µg/kg BW/d from a 2-year gavage study with riddelliine in rats [19]. The HBGV as a preliminary orientation value is quoted with 0.1 µg/kg BW/d (NOAEL/100) for the sum of PA/PANO and should not be exceeded [15].

To assess the acute health risk of adults (70 kg BW) resulting from a short-term exposure of PA/PANO, two exemplary recipes (No. 1: ‘Mediterranean pesto’; No. 2: ‘tomato sauce’; see 4.6) were assumed, including the culinary herbs basil, oregano and thyme. Median and mean contents as well as the 95% percentile (P95) of the sum contents of these matrices were calculated (Table 2).

One portion of the pesto (50 g/adult) resulted in a consumption of 2.5 g of thyme and 3.75 g of oregano and basil each, and for the tomato sauce a consumption of 0.67 g of oregano was estimated (see Section 4.6). In case of two- to five-years aged children (16 kg BW), the 97.5% percentiles (P97.5) of intake of singular raw herbs retrieved from a study on dietary intake of pesticide residues were used [20].

For PA/PANO exposure calculation, mean contents (mean-case scenario) or P95 contents (worst-case scenario) were considered to cover possibly occurring higher PA/PANO contaminations. In order to consider the impact of usage of fresh or dried produce, calculations were performed under application of drying or dehydration factors [21]. The acute health risk was expressed as an exceedance factor of the HBGV of 0.1 µg/kg BW/d (Table 3).

A consumption of one portion of the recipes prepared with mean contaminated, fresh culinary herbs (applying a dehydration factor of 6) resulted in HBGV exceedance factors of 0.1 and 0.3 for adults as well as each 0.2 in case of children. The exceedance factors in the worst-case scenario lay between 0.3 and 1.5 for an adult and 1.1 and 1.2 for a child. When considering dried herbs for the recommended amounts of thyme, basil and oregano in both recipes, adults would exceed the HBGV by 0.3 and 1.7 in the mean-case and 1.7 and 9.3 in the worst-case scenario. In case of children, exceedance factors were 1.2 and 1.3 in the mean-case as well as 6.5 and 7.1 in the worst-case scenario. Due to the high levels within the oregano samples, this matrix was a main contributor for the acute intake of PA/PANO via the presented recipes.

### 2.5. Chronic Health Risk Assessment of PA/PANO in Spices and Culinary Herbs

The chronic, long-term exposure of consumers to PA/PANO was evaluated using the MOE approach based on a BMDL_10_ of 237 µg/kg BW/d [10]. Therefore, the 305 investigated samples were arranged in four groups, with respect to their total PA/PANO contents (Table 4). Means and medians of each risk group were calculated and representative contents for a low, medium and high-risk scenario were derived. The mean contents were chosen for the low and medium-risk group. The rounded median was chosen as representative content for the high-risk group, being the more robust value with respect to the smaller quantity of samples.

The derived representative contents for each risk group were used for calculating MOE values for different mean case and worst-case scenarios for adults and children (Table 5). As no specific intake data on individual spices and culinary herbs were available, the long-term consumption was estimated via the cumulated ingestion of culinary herbs from the two 24 h recalls of the German National Nutrition Survey II [17,22]. In this survey, only data from people of the age 14 to 80 were available, thus in case of children the overall daily intake of herbs reported in [20] was considered for the average consumption scenario. For a high consumption scenario of children, two times the average consumption was assumed. Equally to the acute health risk assessment approach, estimations were performed with respect to dried and fresh produce by applying a dehydration factor of six.

A MOE value > 10,000 is regarded as being of little concern for risk management and public health. When considering fresh herbs for the assumed daily intake (considering a dehydration factor of six), in case of adults a MOE of < 10,000 was only calculated in the high consumption scenario combined with PA/PANO sum levels of 5550 µg/kg (high risk group). Contrary to that, in the case of children, the MOE was below 10,000 in both consumption scenarios when a high contamination of the spices and herbs was assumed. Considering dried herbs for the daily spice and herb consumption and thus no dehydration factors, the results revealed for both adults and children MOE values < 10,000 in every assumed scenario with a high PA/PANO content of 5500 µg/kg. Notably, the high consumption scenario of dried spices and herbs already revealed MOE values < 10,000 for both children and adults when considering a sum content of 330 µg/kg (i.e., medium risk group).

## 3. Discussion

In total, in 178 of the 305 samples (58.4%) at least one PA/PANO was detected. The average content over all 305 samples was 323 µg/kg and the median content was 0.9 µg/kg. The results differed significantly, depending on the investigated spice or herb. Similar findings were recently published: In 2016, the German Federal Institute for Risk Assessment (BfR) investigated 40 spice and herb samples from various matrices and detected a mean content of 265 µg/kg, with amounts up to 4990 µg/kg in a cumin sample [15]. In addition, in 2019 the BfR reported an average sum level of 2680 µg/kg and a median level of 10 µg/kg obtained from a broader study with 263 spice and culinary herb retail samples, covering 17 varieties [17].

Due to the sampling in compliance with Commission Regulation (EC) No. 401/2006, the samples were assumed to be homogenous and representative for a large batch. The other way round, this indicated that PA/PANO levels in retail samples might be even higher, due to the usually occurring spot contamination via parts of PA/PANO containing plants within a bigger spice or herb lot. In consequence, the homogenised samples from the current study might represent lower PA/PANO amounts due to a ‘dilution’ with uncontaminated raw material, whereas individual retail samples could contain very high sum levels due to packaging of contamination ‘spots’ from a large lot. To confirm this hypothesis, more data must be collected in further occurrence studies on retail spice and culinary herb samples. Up to now, only retail samples of oregano were investigated in a prior study [23].

Our findings showed Ht-type PA/PANO as the quantitatively predominant contaminants, whereas Sc-type revealed a higher prevalence. The varying patterns of PA/PANO in the investigated samples indicated that spices and culinary herbs might be contaminated with diverse undesired weeds. Picron et al. reported the presence of PA/PANO in 100% of the 17 investigated spice samples, with contents up to 1770 µg/kg and an average of 197 µg/kg [16]. Interestingly, this study also showed Ht-type PA/PANO to be the main contributors, being responsible for 85% among the total contamination of the study’s samples. These results are in accordance to our findings of Ht-type alkaloids predominantly occurring in affected samples.

Remarkably, even 69% of ginger root samples were found to contain PA/PANO up to 17.8 µg/kg, although ginger is not an herb-based spice and, thus, should normally not be affected by a co-harvesting of contaminant plants. The ginger samples were obtained as dried and milled powder. Indeed, the detected contents seem to be unproblematic, but the findings may indicate a possible cross-contamination during processing in the companies. Another possible way of contamination may be the recently described horizontal transfer of PA/PANO between living plants via the soil [12], especially as ginger is a root spice. In summary, in all 15 spice or herb varieties investigated in the current study, traces of PA/PANO were detected in at least a few samples, indicating PA/PANO to be typical process contaminants.

Our findings showed that, in particular, oregano and cumin often were highly contaminated with PA/PANO. In previous studies, retail oregano was already identified to contain high amounts of PA/PANO. In a study from 2019, the BfR reported an average sum level of 4038 µg/kg and a median level of 942 µg/kg for 59 oregano samples from supermarkets [17]. Kapp et al. investigated 41 retail oregano samples and detected contents higher than 1000 µg/kg in 75% of them, with a mean of 6160 µg/kg, a median of 5430 µg/kg, and a maximum of 32.4 mg/kg [23]. In consequence, our results confirmed these findings, and, in addition, strongly indicated oregano to be one of the main contributors to the overall PA/PANO exposure via the intake of spices and herbs.

Recently, the PA/PANO sum contents in *Senecio vulgaris* L. were determined and shown to range from 0.16% to 0.49% related to dry matter, depending on the developmental stage and season [24]. Compared to the highest content of PA/PANO analytes detected in oregano in our current study (24.6 mg/kg), that indeed mainly originated from Ht-type compounds, this would correspond to a proportion of 1.54% to 0.50% of pure PA/PANO plants in this oregano sample. According to a specification of the European Spice Association (ESA), impurities of extraneous matter of up to 2% are quoted as tolerable for oregano to be marketed as pure oregano [25]. Considering the extremely high contamination levels detected in singular oregano samples in our study, the ESA specification was revealed to be inacceptable when the tolerated 2% extraneous matter were related to toxic plants, for instance PA/PANO containing plants. Thus, the ESA specification may not be seen as generally appropriate.

The maximum levels, which are discussed to be implemented in Regulation (EC) No. 1881/2006 are 400 µg/kg for cumin seeds and other dried herbs as well as 1000 µg/kg for dried marjoram and dried oregano [14]. Considering these limits, four of eleven cumin samples (36%), seven of 24 oregano samples (29%), one of 31 marjoram samples (3%) and ten of 165 other herb samples (6%) would have exceeded the maximum levels. In total, 22 of 231 samples (10%) concerned by the discussed maximum levels would have exceeded them. In conclusion, spices and culinary herbs should be further investigated on their PA/PANO contents. In particular, the results on PA/PANO sum levels of oregano and cumin seemed to be a more extensive problem, originating from the supplying farms itself. Thus, in the course of preventive consumer protection, weed management actions are necessary. In the meantime, trainings of the staff at early production stages were already initiated to tackle the problems of high PA/PANO contents, particularly in oregano.

The risk assessment results revealed the detected sum contents of PA/PANO to be of concern both in view of acute and chronic toxicity. Evaluation of the acute risk using spices and culinary herbs in typical recipes resulted in an exceedance of the HBGV in the worst-case scenario, even when dehydration factors for differences in weight of fresh and dried herbs were taken into account. In consequence, a single ingestion of spices and culinary herbs highly contaminated with PA/PANO may not be seen as safe regarding potential non-neoplastic effects.

Concerning the risk assessment on the chronic intake of PA/PANO via spices and herbs, the presented ingestion scenarios for adults and children resulted in MOE values < 10,000 in some cases, particularly for scenarios considering a high spice and herb contamination of 5500 µg/kg. In conclusion, our findings entirely confirmed former risk assessment data on PA/PANO in freeze-dried herbs published by the BfR in 2019, where exceedance factors of the HBGV of up to 2.1 were calculated [17]. Hereby, the BfR did not consider any dehydration factors.

Indeed, our presented exposure estimations based on short-term and long-term intake of toxic PA/PANO may be afflicted by uncertainties. Two major problems have to be pointed out: the lack of reliable data concerning the consumption of individual spices and herbs, as well as in praxi strongly varying amounts of fresh or dried herbs used in cooking recipes. While reported contents of contaminants usually relate to the dried product, recipes mostly use fresh ingredients. Ignoring dehydration factors can cause a further uncertainty due to overestimations of the consumption of spices and herbs and, thus, of the PA/PANO intake. On the other hand, in case of children the intake was related on raw, unprocessed spices and herbs, and thus consequently lower compared to the data given for processed spices and herbs [20]. In conclusion, our results for risk assessment on the chronic intake of PA/PANO via spices and herbs represent a more conservative estimation, and the true intake might even be higher. In general, the current risk assessment procedure of summing-up the contents of all different types of PA/PANO is subject to uncertainties. This procedure does not respect different toxic potentials of the singular compounds. In order to respect these differences, Merz and Schrenk have proposed relative potency factors (RPF) that may improve the reliability of future risk assessments [26].

Additionally, the calculated content of 5500 µg/kg as representative for the high-risk group (see Table 4) may be regarded as biased, since seven of the ten samples within this group were oregano. On the other hand, the median of the PA/PANO sum content was used for these high risk calculations instead of the mean level. Moreover, more data on the occurrence of PA/PANO in spices and herbs and on their intake by different consumer groups are necessary to improve future risk assessment.

At this point it has to be considered that the intake of PA/PANO estimated in the current study exclusively originated from spices and culinary herbs. Actually, all sources of PA/PANO exposure have to be considered to properly assess the risk of adverse health consequences. In particular honey, (herbal) teas and food supplements are widely known as main contributors for PA/PANO exposure [10]. Formerly published studies on (herbal) teas, honeys and plant-based food supplements already reported MOE values < 10,000 without considering further sources of PA/PANO [27,28,29].

In conclusion, even if our study represents one of the most comprehensive available, there is still a lack of data on the PA/PANO contents in the addressed spice and herb matrices. Regarding single matrices, the number of samples was quite low; geographical and seasonal influences should be addressed in more detail. The presented data concerning the risk for consumers due to consumption of contaminated spices and culinary herbs may be seen as a first indication towards the problem of high contents of PA/PANO in these matrices. Although the total intake of spices and herbs is generally low, the current study undoubtedly revealed that these matrices may significantly contribute to the overall acute and chronic exposure of consumers to toxic PA/PANO.

## 4. Materials and Methods

### 4.1. Chemical Reagents and Standards

The following PA/PANO standards were purchased from PhytoLab (Vestenbergsgreuth, Germany): 7-O-acetylintermedine (AcIm), 7-O-acetylintermedine-N-oxide (AcImN), 7-O-acetyllycopsamine (AcLy), echimidine (Em), echimidine-N-oxide (EmN), erucifoline (Ec), erucifoline-N-oxide (EcN), europine (Eu), europine-N-oxide (EuN), heliotrine (Ht), heliotrine-N-oxide (HtN), intermedine (Im), intermedine-N-oxide (ImN), jacobine (Jb), jacobine-N-oxide (JbN), lasiocarpine (Lc), lycopsamine (Ly), lycopsamine-N-oxide (LyN), monocrotaline (Mc), monocrotaline-N-oxide (McN), retrorsine (Rs), retrorsine-N-oxide (RsN), senecionine (Sc), seneciphylline (Sp), seneciphylline-N-oxide (SpN), senecivernine (Sv), senecivernine-N-oxide (SvN), senkirkine (Sk) and trichodesmine (Td). Other PA standards were obtained from CFM Oskar Tropitzsch (Marktredwitz, Germany), namely 7-O-acetyllycopsamine-N-oxide (AcLyN), indicine (Ic), indicine-N-oxide (Ic), integerrimine (Ig), integerrimine-N-oxide (IgN), jacoline (Jl), jacoline-N-oxide (Jl), lasiocarpine-N-oxide (LcN), merenskine (Mk), merenskine-N-oxide (MkN), merepoxine (Mx), merepoxine-N-oxide (MxN), sceleratine (Sl), sceleratine-N-oxide (SlN) and senecionine-N-oxide (ScN).

With respect to solubility, stock solutions (c = 1 mg/mL) of each PA/PANO were prepared, either with acetonitrile (Em, Ec, EcN, Eu, Ht, Ic, IcN, Jb, Lc, LcN, Ly, Mc, SpN, Sk) or acetonitrile/water (50/50, v/v) (all other analytes), and stored at 6 °C in the dark. A PA/PANO mix solution (c = 10 µg/mL of each analyte) was prepared by combining stock solution aliquots and diluting the resulting mixture with acetonitrile/water (50/50, v/v). Acetonitrile and methanol (both LC-MS grade) were used for all experiments and purchased from Th. Geyer (Renningen, Germany). Ultrapure water was obtained using an UltraClearTM TP UV UF TM system from Evoqua Water Technologies (Barsbüttel, Germany). Sulphuric acid and formic acid were purchased from Th. Geyer (Renningen, Germany). Ammonia was purchased from Merck (Darmstadt, Germany), and ammonium formate, used as an additive for LC-MS solvents, was obtained from Fluka (Steinheim, Germany).

### 4.2. Sampling

In total, 305 samples were provided by German spice and herb suppliers associated to the Association of the German Spice Industry (Table S1). The samples covered 15 spice and culinary herb matrices originating from 36 countries. They were harvested within the years 2014 to 2018, with a majority of samples originating from 2016 and 2017. Where possible, sampling was conducted according to European Commission Regulation No. 401/2006 concerning sampling and analysis of mycotoxins in foodstuff [30]. All samples were lyophilized and thus the weight of samples taken relied exclusively on dry matter.

### 4.3. Sample Pre-Treatment and Extraction

Each sample was homogenized to a particle size < 1 mm using a ZM 200 centrifugal mill from Retsch (Haan, Germany) and stored at 20 °C at a dry and dark place until analysis. Two independent biological replicates were generated from each sample according to an already described extraction procedure [18]: In brief, 40 mL of sulphuric acid (0.05 mol/L) were added to 2.0 g of dry sample material and vortexed, treated in an ultra-sonic bath (10 min) and horizontally shaken (500 U/min, 20 min). After centrifugation for 10 min at 5000× *g*, the raw sample extract was filtered through a folded filter and 10 mL were loaded on a solid phase extraction (SPE) cartridge (Agilent Bond Elut Plexa PCX, 500 mg/6 mL, Santa Clara, CA, USA), preconditioned with 5 mL each of methanol and sulphuric acid (0.05 mol/L). After washing with 5 mL of water and 5 mL of methanol, the analytes were eluted into a glass vial using 10 mL of ammoniated methanol (5%). The eluates were dried under nitrogen at 50 °C and reconstituted in 1.0 mL of LC solvent A (water containing 0.1% formic acid and 5 mmol/L ammonium formate), shaken with a vortex laboratory shaker, and filtered into a glass vial using a 0.45 µm PVDF syringe filter (Berrytec, Grünwald, Germany).

### 4.4. LC-MS/MS Instrumentation and Software

For all measurements, a Shimadzu high performance liquid chromatography (HPLC) apparatus, including binary pumps, degasser, autosampler, column oven and control unit (LC-20AB, SIL-20AC HT, CTO-20AC, CBM-20A, Duisburg, Germany) was used. The HPLC was coupled to an API4000 triple quadrupole mass spectrometer (MS) from Sciex (Darmstadt, Germany). MS ion source parameters were set as follows: ionization voltage, 2.500 V; nebulizer gas, 50 psi; heating gas, 50 psi, curtain gas, 30 psi, temperature, 600 °C; collision gas level, 7. For data acquisition and processing, Analyst (Version 1.6.2, Sciex, Framingham, MA, USA) and MultiQuant software (Version 3.0.1, Sciex, Framingham, MA, USA) were used. Figures were drawn using OriginPro software (Version 2019, OriginLab, Northampton, MA, USA).

### 4.5. Measurements and Quantification

The measurements were conducted according to Kaltner et al. [18]: A 150 × 2.1 mm Kinetex^TM^ 5 µm CoreShell EVO C18 100 Å column protected by a SecurityGuard™ ULTRA EVO C18 2.1 mm guard column (both Phenomenex, Aschaffenburg, Germany) was used for chromatographic separation of PA/PANO analytes. Used solvents were water (A) and acetonitrile/water (95/5, v/v, B), each containing 0.1% formic acid and 5 mmol/L ammonium formate. The column oven temperature was maintained at 30 °C and 20 µL of sample extract were injected. The binary linear gradient conditions at a flow rate of 0.4 mL/min were: 0.0 min 2% B, 10.0 min 9.5% B, 15.0 min 39% B, 15.1 min 100% B, 16.5 min 100% B and an additional re-equilibration of 6.5 min prior to each run.

The quantification was performed via external matrix matched calibration, or, in case of chive, curry and herbs of Provence samples, via standard addition, respectively. Aliquots of the PA/PANO mix standard solution were pipetted into glass vials, dried under nitrogen at 50 °C and reconstituted with extracts of a suitable blank sample to prepare calibration standards at concentrations of 0, 1.0, 5.0, 10.0, 25.0 and 50.0 ng/mL. MultiQuant software was used to calculate the respective concentrations in a sample vial applying linear regression on the calibration standards’ peak areas. The matrix matched calibration standards were freshly prepared for each measurement day. LOD ranged from <0.1 µg/kg (LcN) to 2.6 µg/kg (Mk). If the content calculated for an individual compound was between the LOD and the limit of quantification (LOQ), it was calculated with 0.5 times the LOQ. Amounts smaller than the LOD were considered as ‘0.0 µg/kg’ for calculation reasons. Overall, recoveries ranged from 50% (Sp) to 119% (Im) for 40 of 44 PA/PANO compounds. All quantitation results were related to dry matter and were not corrected for recovery rates.

### 4.6. Risk Assessment

For the evaluation of the risk of a short-term (acute) intake of PA/PANO from spices and culinary herbs, two exemplary recipes containing herbs were assumed. Recipe 1 ‘Mediterranean pesto’ (https://www.kraeuter-buch.de/magazin/schmackhaftes-kraeuterpesto-zum-selber-machen-35.html, accessed on 15 January 2020) contained 10 g of thyme as well as 15 g of oregano and basil in 250 mL/200 g of the final pesto, resulting in 2.5 g of thyme and 3.75 g of oregano and basil per portion of 50 g pesto. Recipe 2 ‘tomato sauce’ originated from a recipe book and was also used by the BfR in an earlier study [17]. Here, 2 g of oregano were assumed to be used for the whole recipe (= 3 portions), resulting in 0.67 g of oregano per portion. For comparison reasons, children were estimated to consume the same culinary herbs used in both recipes, but the ingested amounts of the singular herbs were taken form a special nutrition study for two- to five-years aged children [20]. Within this list, the consumption data of raw, unprocessed spices and herbs were used.

The risk of an acute non-carcinogenic health damage was assessed on the basis of a HBGV which has been derived from a NOAEL of 10 µg/kg BW/d from a 2-year gavage study with riddelliine in rats [19]. The HBGV as a preliminary orientation value was quoted with 0.1 µg/kg BW/d (NOAEL/100) for the sum of PA/PANO. The median and mean contents as well as the 95% percentile (P95) of the sum contents of the herbs basil, oregano and thyme were used (see Table 2).

For evaluating the risk of a long-term (chronic) PA/PANO intake, the MOE approach was used, based on a BMDL_10_ of 237 µg/kg BW/d for the sum of PA/PANO intake. Therefore, all 305 investigated samples were grouped into four subgroups, according to their PA/PANO sum contents: <LOD, low content (LOD—100 µg/kg), medium content (100–1000 µg/kg) and high content (>1000 µg/kg). The means and medians of each group were calculated, rounded and used as representative contents for a low, medium and high-risk contamination group. In case of adults, data on the consumption of spices and culinary herbs (in g/kg BW/d) were taken from Table 3 from [17]; originally derived from the German National Nutrition Survey II [22]. For children, an average daily intake of herbs of 0.7 g was taken into account [20], and for the high consumption scenario of children, two times the average intake was suggested.

Consumption of spices and culinary herbs for the calculation of both the risk due to acute and the chronic exposure were related to an adult of 70 kg BW as proposed by EFSA [31]. In case of children, data were related to 16 kg BW as proposed by [20].

To consider the use of fresh or dried herbs for the exemplary recipes and the long-term intake of PA/PANO, the calculations were conducted with and without dehydration factors, respectively, as recommended by the ESA [21].

## Figures and Tables

**Table 1:** Sum contents, origins and matrices of the 24 samples with pyrrolizidine alkaloids (PA) and PA *N*-oxides (PANO) sum contents above the currently discussed maximum level of 400 µg/kg [14]. The predominant PA/PANO and their quantitative contribution to the sum contents are added.

PA/PANO Sum Content (µg/kg)	Place of Origin	Sample Type	Predominant PA/PANO	Contribution (% of Sum Content)
24,600	Turkey	Oregano	Europine-NO	82
18,100	Turkey	Oregano	Europine-NO	70
11,000	Greece	Oregano	Europine-NO	42
8300	Turkey	Oregano	Europine-NO	74
6800	Turkey	Oregano	Europine-NO	86
4310	Turkey	Cumin	Europine-NO	36
3300	Germany	Parsley	Senecionine-NO	31
1820	Turkey	Oregano	Europine	50
1780	Greece	Oregano	Europine-NO	55
1110	Albania	Savory	Lycopamine-NO	61
861	Turkey	Oregano	Lycopamine-NO	57
859	Albania	Savory	Lycopamine-NO	35
737	Germany	Parsley	Senecionine-NO	26
709	Albania	Savory	Lycopamine-NO	57
681	Tunisia	Dill	Lasiocarpine-NO	59
666	Unknown	Savory	Lycopamine-NO	63
661	Croatia	Savory	Lycopamine-NO	32
658	Germany	Parsley	Senecionine-NO	43
648	Poland	Oregano	Senecionine-NO	47
540	Germany	Chive	Erucifoline-NO	48
536	Turkey	Cumin	Europine-NO	40
524	Egypt	Marjoram	Senecionine-NO	34
493	Turkey	Cumin	Heliotrine	38
410	Turkey	Cumin	Heliotrine-NO	43

NO = *N*-oxide.

**Table 2:** Number of samples and positive samples, median, mean and 95% percentiles (P95) of pyrrolizidine alkaloids (PA) and PA *N*-oxides (PANO) contents in culinary herbs selected for an acute health risk assessment.

Spice/Herb	*n*	Positive Samples	Median (µg/kg)	Mean (µg/kg)	P95 (µg/kg)
Basil	25	8	0.0	11.5	68.3
Oregano	24	21	163	3140	17,000
Thyme	29	18	4.3	49.1	191
All samples	305	178	0.9	323	665

**Table 3:** Mean- and worst-case scenario for short-term (acute) exposure to pyrrolizidine alkaloids (PA) and PA *N*-oxides (PANO) due to consumption of two exemplary recipes containing culinary herbs. Intake by children were considered as given in [20]. With respect to consumption of fresh or dried herbs, exceedance factors were calculated with or without a dehydration factor (DF) of six, according to [21].

Recipe	Herb	BW^1^ (kg)	Consumption (One Portion/d)		PA/PANO Content	PA/PANO-Intake	Exceedance Factor^4^	
			(g)	(g/kg BW)	(µg/kg)	(µg/kg BW)	no DF	DF = 6
					Mean case^5^			
Medi-terranean pesto	Thyme	702	2.5	0.036	49.1	0.002		
	Oregano		3.75	0.054	3140	0.17		
	Basil		3.75	0.054	11.5	0.001		
						0.173	1.7	0.3
	Thyme	163	1.1	0.069	49.1	0.003		
	Oregano		0.6	0.038	3140	0.119		
	Basil		11.1	0.694	11.5	0.008		
						0.13	1.3	0.2
Tomato sauce	Oregano	702	0.67	0.01	3140	0.031	0.3	0.1
	Oregano	163	0.6	0.038	3140	0.119	1.2	0.2
						Worst case^6^		
Medi-terranean pesto	Thyme	702	2.5	0.036	191	0.007		
	Oregano		3.75	0.054	17,000	0.918		
	Basil		3.75	0.054	68.3	0.004		
						0.929	9.3	1.5
	Thyme	163	1.1	0.069	191	0.013		
	Oregano		0.6	0.038	17,000	0.646		
	Basil		11.1	0.694	68.3	0.047		
						0.706	7.1	1.2
Tomato sauce	Oregano	702	0.67	0.01	17,000	0.17	1.7	0.3
	Oregano	163	0.6	0.038	17,000	0.646	6.5	1.1

^1^ BW = body weight; ^2^ Adult, 70 kg BW; ^3^ child, 16 kg BW; ^4^ related to a health-based guidance value (HBGV) of 0.1 µg/kg BW/d; ^5^ mean levels of PA/PANO contamination; ^6^ 95% percentile levels of PA/PANO Contamination.

**Table 4:** Number of samples classified in several risk groups depending on their sum contents of pyrrolizidine alkaloids (PA) and PA *N*-oxides (PANO). Median and mean contents were calculated for each risk group and representative contents were derived according to the approach applied in [17].

Group	*n*	%	Median (µg/kg)	Mean (µg/kg)	Representative Contents (µg/kg)
<Limit of detection (LOD)	127	41.6	n.c.	n.c.	n.c.
Low risk (LOD-100 µg/kg)	124	40.7	9.2	21.1	20
Medium risk (100–1000 µg/kg)	44	14.4	236	332	330
High risk (>1000 µg/kg)	10	3.3	5550	8120	5500

n.c., not calculable.

**Table 5:** Margin of Exposure (MOE) values resulting for different average and high herb consumption scenarios for both adults and children. The considered sum contents of pyrrolizidine alkaloids (PA) and PA *N*-oxides (PANO) were derived from different risk groups derived from results of the 305 investigated spice and culinary herb samples (Table 4). With respect to consumption of fresh or dried herbs MOE values were calculated with or without a dehydration factor (DF) of six, according to [21].

Scenario	Consumption^1^ (g/d) (g/kg BW/d)		PA/PANO Content^2^ (µg/kg)	Intake (µg/kg BW/d)	Margin of Exposure^3^	
					no DF	DF = 6
Adult, 70 kg average consumption (median)	0.77	0.011	20	< 0.000	n.c.	n.c.
			330	0.004	59,300	356,000
			5500	0.061	3890	23,300
Adult, 70 kg Average consumption (mean)	1.68	0.024	20	< 0.000	n.c.	n.c.
			330	0.008	29,600	178,000
			5500	0.132	1800	10,800
Adult, 70 kg high consumption (P95)	6.09	0.087	20	0.002	119,000	711,000
			330	0.029	8170	49,000
			5500	0.479	495	2970
Child, 16 kg average consumption (mean)	0.7	0.044	20	0.001	237,000	1,420,000
			330	0.015	15,800	94,800
			5500	0.242	979	5880
Child, 16 kg high consumption (2x average)	1.4	0.088	20	0.002	119,000	711,000
			330	0.029	8170	49,000
			5500	0.481	492	2950

^1^ Based on [20,22]; ^2^ see Table 4; ^3^ Based on a Benchmark Dose Lower Confidence Limit 10% (BMDL_10_) of 237 µg/kg BW/d for the sum of PA/PANO intake. n.c., not calculable.

## Supplementary Materials

The following what are available online at https://www.mdpi.com/2072-6651/12/3/155/s1, Table S1: Contents of pyrrolizidine alkaloids (PA) and PA-*N*-oxides in 305 investigated spice and culinary herb samples.
